# First Detection of Bluetongue Virus Type 3 in Poland in 2024—A Case Study in European Bison (*Bison bonasus*)

**DOI:** 10.3390/pathogens14040377

**Published:** 2025-04-12

**Authors:** Magdalena Larska, Anna Orłowska, Wojciech Łopuszyński, Łukasz Skurka, Agnieszka Nowakowska, Paweł Trębas, Michał K. Krzysiak, Jerzy Rola, Marcin Smreczak

**Affiliations:** 1 Department of Virology and Viral Animal Diseases, National Veterinary Research Institute, Al. Partyzantów 57, 24-100 Puławy, Poland; anna.orlowska@piwet.pulawy.pl (A.O.); agnieszka.nowakowska@piwet.pulawy.pl (A.N.); p.trebas@piwet.pulawy.pl (P.T.); jrola@piwet.pulawy.pl (J.R.); 2 Department of Pathomorphology and Forensic Veterinary Medicine, Faculty of Veterinary Medicine, University of Life Sciences, Głęboka 30, 20-612 Lublin, Poland; wojciech.lopuszynski@up.lublin.pl (W.Ł.); michal.krzysiak@up.lublin.pl (M.K.K.); 3 Department of Parasitology and Fish Diseases, Faculty of Veterinary Medicine, University of Life Sciences, Akademicka 12, 20-950 Lublin, Poland

**Keywords:** bluetongue virus, BTV-3, Europe, wildlife, European bison, *Culicoides* spp.

## Abstract

Since the emergence of serotype BTV-3, another bluetongue virus, in fall 2023, this variant has been causing great losses in livestock farming in Europe. The virus spreads faster than the epidemic BTV-8, which appeared on the continent nine years earlier. This study describes the first case of BTV-3 in Poland detected in a European bison (*Bison bonasus*) in Poland’s Wolin National Park, approximately 15 km from the German–Polish border. The animal suffered from a severe and fatal hemorrhagic disease. The symptoms included respiratory problems, bloody diarrhea, and rapidly progressive cachexia. In addition to the virus’s confirmation as BTV-3 and the presence of the infecting agent in the blood and spleen of the animal, the virus was also detected in one pool of blood-fed *Culicoides punctatus* caught near the bison enclosure two weeks after the death of the bison. This is the first detection of BTV-3 in *C. punctatus*, which suggests vector competency for this serotype. Phylogenetic analysis based on segment 2 of the virus revealed the homology of the Polish isolate to the BTV-3 strains circulating in the Netherlands, Germany, and Portugal, and slightly lower similarity to the BTV-3 strains detected in sheep in Sardinia (Italy) in 2018 and in Tunisia in November 2016. A retrospective serosurvey of the exposure to BTV in thirteen other European bison populations distributed over the country indicated that the observed case at the Wolin National Park was the first BTV-3 to be detected in Poland.

## 1. Introduction

The role of wildlife in the transmission and maintenance of many pathogens in the environment remains highly understudied. Emerging climate-sensitive infections (CSIs)—an example of which is bluetongue (BT) (caused by a virus of the same name)—are spread across borders and transcontinentally via vectors whose range and activity are changing with global warming. Bluetongue virus (BTV) is an orbivirus in the Reoviridae family and is known to affect domestic and wild ruminants and camelids. It is transmitted by nocturnal blood-sucking midges of the *Culicoides* genus. Historically, BTV circulation was limited to the tropical and subtropical climates of Africa, but since the mid-20th century it has also been circulating in the Mediterranean basin and the Americas. The expansion was believed to be related to the competence of the vectors *C. imicola* in the Mediterranean and *C. sonorensis* in the Americas. The virus is considered predominantly pathogenic to sheep, but as it occurs in 24 different “classical” serotypes and several novel serotypes referred to as atypical, these may differ in their tropism and virulence to vertebrate hosts. Interestingly, BTV infections are considered subclinical in tropical zones, while the introduction of susceptible animals into BTV-endemic areas results in clinical disease emergence in temperate zones [1]. The first major intrusion of BTV serotype 8 into central Europe above latitude 50° N in 2006 caused havoc in the animal production sector and forced the reassessment of epidemic risks, particularly in terms of climate change [2]. Now, almost two decades later, new BTV serotypes are often reported in Europe, and the latest to spread from the center of the continent, doing so at a record rate, is BTV-3 [3,4,5,6,7,8]. The rapid contemporary expansion of, often, tropical or neglected diseases in Europe is also linked to a wide range of reservoir species that the agents infect or through which they are able to adapt to and acquire the possibility of overwintering within while remaining endemic in the area. Vertical transmission of BTV in *Culicoides* spp. has not been demonstrated to be responsible for inter-seasonal virus transmission [9,10], as has been suggested for *Culicoides*-borne Schmallenberg virus (SBV) [11]. Therefore, it is very likely that wildlife plays an important role in the maintenance of BTV in the environment [12,13]. The omission of wildlife from official BTV monitoring or eradication programs likely leads to an underestimation of the prevalence of these CSIs.

The European bison, or wisent, (*Bison bonasus*) is the largest wild ruminant in Europe. It became extinct in the wild in north-eastern Poland (the lowland European bison) and the Caucasus in Russia (the Caucasian European bison) after the First World War. The species was returned to the wild; however, it remains protected by the International Union for Conservation of Nature and is categorized as ‘near threatened’ on the Red List of Threatened Species [14]. Nearly a century has gone by since the start of the restitution breeding of *Bison bonasus* in the Białowieża Forest in Poland, and the European population of the species in 2023 exceeded 11,000 individuals, almost 80% of which remain free-ranging [15]. Most European bison live in Poland, Russia, and Belarus (Figure S1). Quite numerous and still-thriving populations also exist in Germany, Romania, Ukraine, and Lithuania. European bison have even been introduced into the Iberian peninsula, despite local discussions regarding the allochthonous nature of the species [16]. Evidence of the onetime presence of these emblematic large herbivores in northern Spain and southern France are prehistoric remains and paintings, such as those found in the Altamira cave, Cantabria, Spain, of the European bison’s larger analog, the steppe bison (*Bison priscus*). Climate change forced such megafauna to migrate to the northern hemisphere, where they became extinct thousands of years ago. The observations presented here shed some light on two scenarios of exposure risk: when progressive environmental changes threaten native fauna, and when animals are exposed to threats via human activity, globalization, and relocation of animals to drastically different environments. It is interesting to note that this is the first case of BTV-3 infection in Poland, and it was detected a few weeks before any other cases were found by national active monitoring in subclinically infected cattle. In addition to the first description of the clinical picture of BTV-3 infection and the pathological changes caused by it in the European bison, we present the details of entomological monitoring in its sylvatic habitat, the identification of the virus in a *Culicoides* vector, and an analysis of the BTV epizootic situation in fourteen European bison populations between 2023 and 2024.

## 2. Materials and Methods

### 2.1. Case Study and Clinical Diagnosis

**Figure 1:**
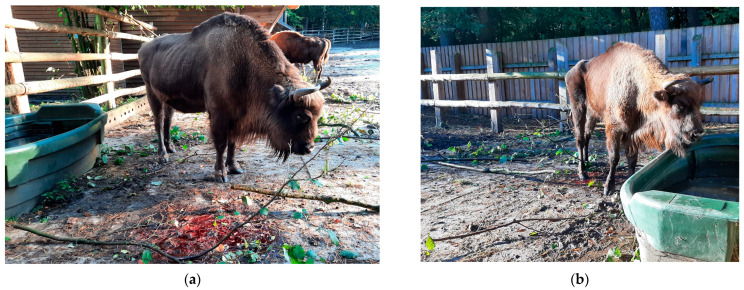

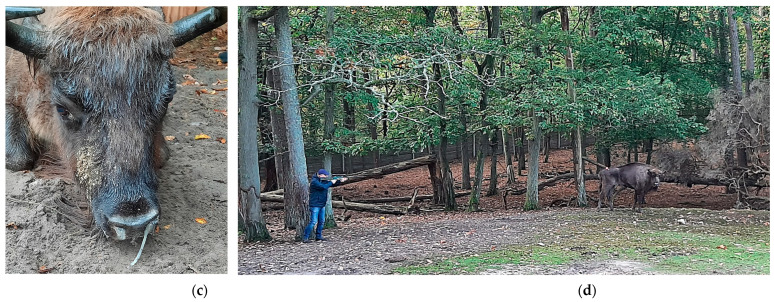
A case of bluetongue in a 9-year-old European bison cow in an enclosure of Wolin National Park, Poland: (**a**) bloody diarrhea, reduced appetite, and lethargy; (**b**) bloody diarrhea, loss of appetite, impaired water intake, and gradual emaciation; (**c**) nasal discharge and stupor; (**d**) drug administration by dart from an air rifle.

The death of a 9-year-old female European bison was reported on 15 October 2024 in the European bison game reserve at Wolin National Park (WNP; N 53°56′2.107′′ E 14°28′34.402′′), which is located on an island in the Baltic Sea in the immediate vicinity of Germany’s eastern border. Approximately 10 days prior to her death, the animal had suffered a gradual deterioration of health and lack of appetite and had separated herself from the herd. In the last 2 or 3 days, the animal developed bloody diarrhea, serous and then purulent nasal discharge, debilitation, apathy, and impaired mobility (Figure 1a–c). Treatment was given with Draxxin, Baytril, and Naxel injected intramuscularly by dart gun (Figure 1d) and intravenous infusions of Ringer’s lactate solution, 40% glucose, Biotyl, Duphalyte, and Meloven, while the bison was already unable to stand. A postmortem examination was performed on-site no more than a few hours after her death, and samples of the parenchymal organs, intestines, blood in EDTA, and clotted blood were sent, chilled to 4 °C, to the laboratory of the National Veterinary Research Institute in Poland for virological and bacteriological testing. Fragments of the lungs, liver, spleen, kidneys, and small and large intestines were fixed in 10% formalin at pH 7.2 for histopathological examination. The tissues were then dehydrated in alcohol solutions, acetone, and xylene in a tissue processor (Leica TP-1020; Leica Biosystems, Nussloch, Germany) before being embedded in paraffin blocks. For the analysis, 4 μm-thick tissue sections were stained with hematoxylin and eosin (HE) and observed under a microscope.

Some transient nasal discharge and moderate depression were observed in another two European bison in the herd; however, the symptoms quickly resolved without the need for veterinary intervention. Sampling was not undertaken due to the cost and risk associated with the necessary chemical immobilization of the animals.

### 2.2. Culicoides Monitoring

**Figure 2:**
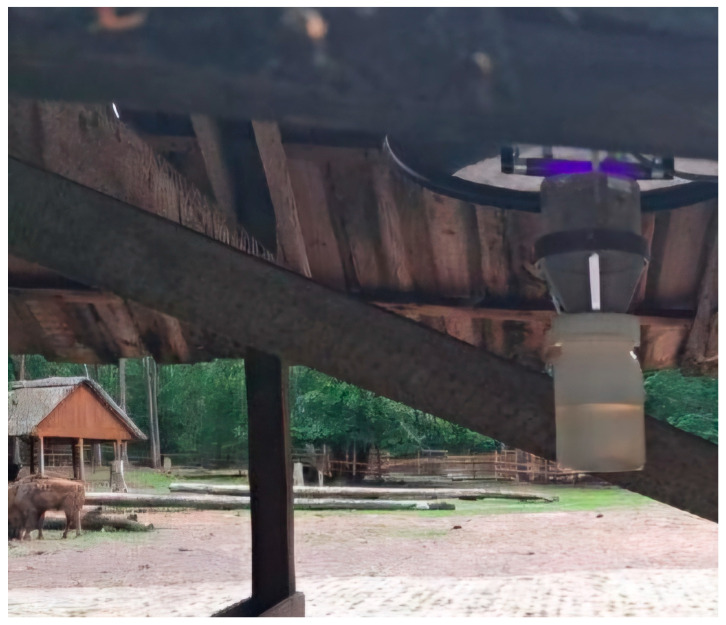
A CDC 1212 UV light trap (John W. Hock Company, Gainesville, FL, USA) for *Culicoides* spp. collection installed at the European bison enclosure of Wolin National Park, Poland, in 2024.

Due to the increasing threat of BTV and epizootic hemorrhagic disease virus transmission from the west of Europe, *Culicoides* monitoring also included the WNP area in 2024. Entomological studies were conducted, as described previously [11], on the basis of WNP consent no. 42.20.1.2024. The insects were collected using an ultraviolet light trap (CDC 1212, John W. Hock Company, Gainesville, FL, USA) located in the immediate proximity of the European bison enclosure (Figure 2), which was set up every one or two weeks overnight from 16 June until the end of the annual activity of these nocturnal insects (2 December). The midges, which were attracted by UV light, were sucked in by a fan and finally trapped in the container with sterile water and a drop of detergent. The insects were sieved and placed in 70% ethanol until testing under an SDF PLAPO 1XPF objective in an SZX16 microscope (Olympus, Tokyo, Japan). Entomological examination began with species identification based on the methodology described by Mathieu et al. [17]. The examination continued with the determination of sex and female gonotrophic cycle stage (a virgin, unpigmented abdomen being indicative of nulliparous females; a pigmented, blood-filled abdomen indicating a female is parous, having reproduced at least once; an abdomen filled with eggs batches classifying the midge as gravid; and visible signs of a recent meal indicating a blood-fed individual). The final entomological examination stage involved the preparation of pools of up to 20 blood-fed individuals for molecular testing.

### 2.3. Surveillance Sampling

**Figure 3:**
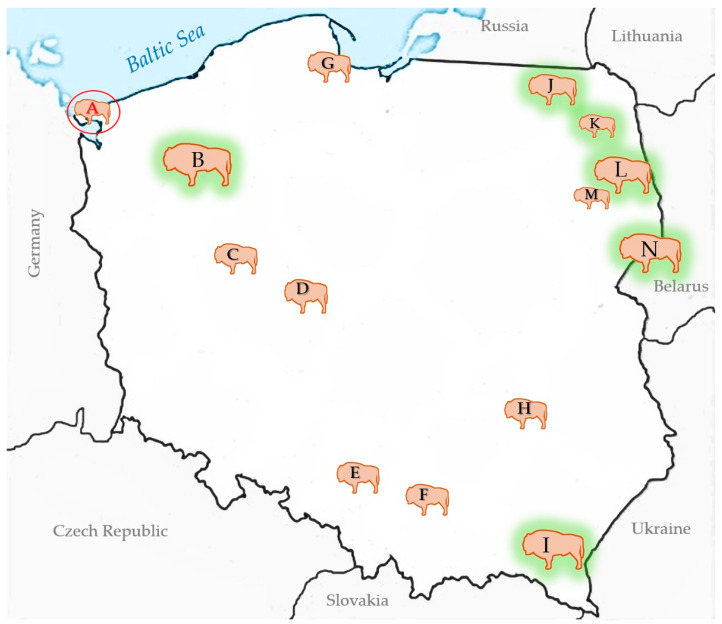
Distribution of the European bison populations in Poland. The free-ranging populations are marked with green halos. The location of the index case at Wolin National Park—A—is indicated with a red oval.

Surveillance was carried out as part of a conservation strategy for the species, as described previously [18,19]. For monitoring purposes, 160 European bison serum samples collected between 2023 and 2024, prior to the occurrence of the case detected at WNP, were retrospectively tested for the presence of BTVspecific antibodies. In the case of a seropositive animal, a virological examination of the full-blood sample was performed. The animals originated from seven free-ranging populations, including the three largest ones in Europe, namely the Białowieża Forest (N), Bieszczady Mountain (I), and Zachodniopomorskie herds (B); and seven captive herds spread across the country (Figure 3).

The samples originated from both female (*n* = 71) and male (*n* = 89) European bison aged between 2 days and 23 years. The European bison were sampled solely when other procedures were performed and were not immobilized or euthanized for the tests described here. Most samples were taken from clinically healthy bison, which were pharmacologically immobilized to be fitted with collars with telemetric transmitters, or for the mandatory testing of translocated individuals (*n* = 84). Some samples were collected from fallen bison, meaning individuals which were found dead (*n* = 25). Other samples were from bison euthanized (*n* = 40) due to poor health in accordance with the corresponding decisions of the Minister of the Environment and the General Director for Environmental Protection, or from bison which died after being struck by cars or trains (*n* = 7). From the immobilized or recently eliminated animals, the blood was collected through a puncture either of the external jugular vein (*vena jugularis externa*), or less often, from the tail vein (*vena caudalis mediana*). Blood from necropsied animals was collected in the form of a clot from the heart or from body cavities. Blood was collected into sterile 9 mL EDTA tubes and serum clot activator tubes, which were centrifuged within 24 h. The full-blood and serum samples were stored at −70 °C in the biobank of the Department of Virology and Viral Animal Diseases at the National Veterinary Research Institute until analysis.

### 2.4. Virological Testing

#### Virus Isolation

Bluetongue virus was isolated in a baby hamster kidney clone 21 (BHK-21) cell line in accordance with the SOP of the European Reference Laboratory for Bluetongue in Algete, Spain. Monolayers of BHK-21 cells were overlaid with blood cells extracted from EDTA-treated whole blood after the removal of virus-neutralizing antibodies, and with a 20% homogenate (*w/v*) of spleen prepared in Eagle’s minimum essential medium. The cell cultures were incubated at 37 °C in 5% CO_2_ with humidity. The removal of virus-neutralizing antibodies was performed according to a World Organization for Animal Health protocol [20]. The cell monolayers were monitored microscopically for the appearance of cytopathic effects (CPEs) for up to 5–7 days. If no CPE was observed, subsequent passage in the BHK-21 cell culture was performed. Positive CPE or negative cell culture results for BTV isolation were confirmed after each cell culture passage by real-time RT-PCR testing.

### 2.5. Molecular Testing

#### 2.5.1. Nucleic Acid Extraction

Nucleic acids were extracted from the whole blood and 10% homogenate of spleen tissue collected intravitally or postmortem from European bison and from pools of blood-fed *Culicoides.* Tissue and insect samples were mechanically homogenized in phosphate-buffered saline (PBS) with 1.4 mm ceramic (zirconium silicate) beads (Lysing Matrix D, MP Biomedicals, Irvine, CA, USA) using a TissueLyser LT (Qiagen) and two cycles of 45 s at 6500 rpm, with an interval of sample cooling on ice, as described previously [11]. Homogenates were clarified at 2500 rpm for 5 min at 4 °C. Total nucleic acid extraction was performed from 200 mL of the EDTA blood sample, spleen homogenate, or insect suspension supernatant using an IndiMag Pathogen Kit (Indical, Leipzig, Germany) in an IndiMag 48s machine for automated nucleic acid extraction, following the manufacturer’s protocol. Nucleic acids were used immediately for RT-PCR or were preserved at −20 °C until use.

#### 2.5.2. RT-PCR

Detection of BTV RNA was conducted in a pan-BTV real-time RT-PCR as described by Hofmann et al. [21]. The reaction targeted the NS3 segment fragment, according to the European Union Reference Laboratory’s BT standard operating protocol and the WOAH’s recommendation [20]. Briefly, 2 µL of RNA and 10 µM of oligonucleotides were denatured at 95 °C for 5 min and immediately cooled on ice for 3 min. Next, a mixture of 0.2 uM BTV probe, 12.5 µL of 2 × RT-PCR buffer, 8 µM of oligonucleotides for mammalian β-actin detection or 18 S rRNA of *Culicoides* as an internal control (housekeeping gene), and enzyme mix were added. The real-time RT-PCR was performed with a QuantStudio 6 instrument (Applied Biosystems/Thermo Fisher Scientific, Foster City, CA, USA). Samples were classified as positive if the threshold cycle (C_t_) value was lower than 40. Samples with a C_t_ greater than or equal to 38 were considered doubtful and retested. For BTV-3 typing, a commercial Adiavet BTV Type 3 kit was used. Typing was carried out according to the instructions of the manufacturers of the Adiavet kit and the QuantStudio 6 instrument. Phylogenetic studies followed BTV-3 typing, for which a gel-based RT-PCR was designed to target and detect a 1000 bp fragment of segment 2 of BTV-3. The primer sequences were as follows: BTV_3_AO_F: 5′-AATYACCTATTYAATACCGC-3′ and BTV_3_AO_R: 5′-TCATCTCACGATATCTATC-3′. The PCR products were visualized in 2% horizontal electrophoresis agarose gel, and after purification were subjected to automated Sanger sequencing in two directions on an ABI PRISM 310 Genetic Analyzer (Applied Biosystems/Thermo Fisher Scientific) using a BigDye Sequencing Kit with GeneScan Analysis Software (both from Applied Biosystems/Thermo Fisher Scientific).

#### 2.5.3. Phylogenetic Studies

Nucleotide sequences of a 1000 bp fragment of segment 2 of BTV were aligned using Clustal W Multiple alignment, and a phylogenetic neighbor-joining tree was generated using an appropriate evolutionary model bootstrapped on the set of 1000 replicates with MEGA 5 software [22]. The similarity matrix was derived using the BLOSUM62 matrix in BioEdit software v. 7.0.5.3.

### 2.6. Serological Testing

An INgezim BTV DR (double-recognition) 12.BTV.K0 ELISA (INGENASA, Madrid, Spain) targeting VP7 protein-specific antibodies of 24 BTV serotypes was used in accordance with the instructions provided by the manufacturer. The kit is intended for testing bovine, sheep, or goat serum samples. Briefly, 50 µL of serum was diluted with 50 µL of diluent and mixed. The plate was sealed and incubated for 1 h at 37 °C and washed 6 times (300 µL/well) using washing solution previously diluted to a ratio of 1:24 in deionized water. A 100 µL volume of ready-to-use peroxidase conjugate was added to each well, and the plate was sealed and incubated for 1 h at 37 °C. After the plate was rinsed with washing solution, 100 µL of 3,3′,5,5′-tetramethylbenzidine substrate was added to each well, and the plate was sealed again and incubated for 15 min at room temperature. After this step, 100 µL of stop solution was added to each well. The optical density (OD) was read at 450 nm. Samples were considered positive if the OD was higher than the cut-off (15% of positive control) and negative if the OD value was equal to or lower than the positive cut-off. The specificity and sensitivity of this ELISA test were 100% and 99.8%, respectively, according to the manufacturer.

### 2.7. Additional Testing

During the investigation into the cause of death, a number of other tests were performed, including serological assays for antibodies specific to alphaherpesvirus (bovine herpesvirus type 1), pestivirus (bovine viral diarrhea virus), Schmallenberg virus, and bovine coronavirus (BCoV), according to the methods previously described [18]. The nested PCR described by VanDevanter et al. [23] was adopted for the detection of gammaherpesvirus, including malignant catarrhal fever virus. Culture tests were also carried out for aerobic and anaerobic bacterial infections in internal organ tissues and the small intestine.

## 3. Results

### 3.1. Case Study

#### 3.1.1. Necropsy Findings

**Figure 4:**
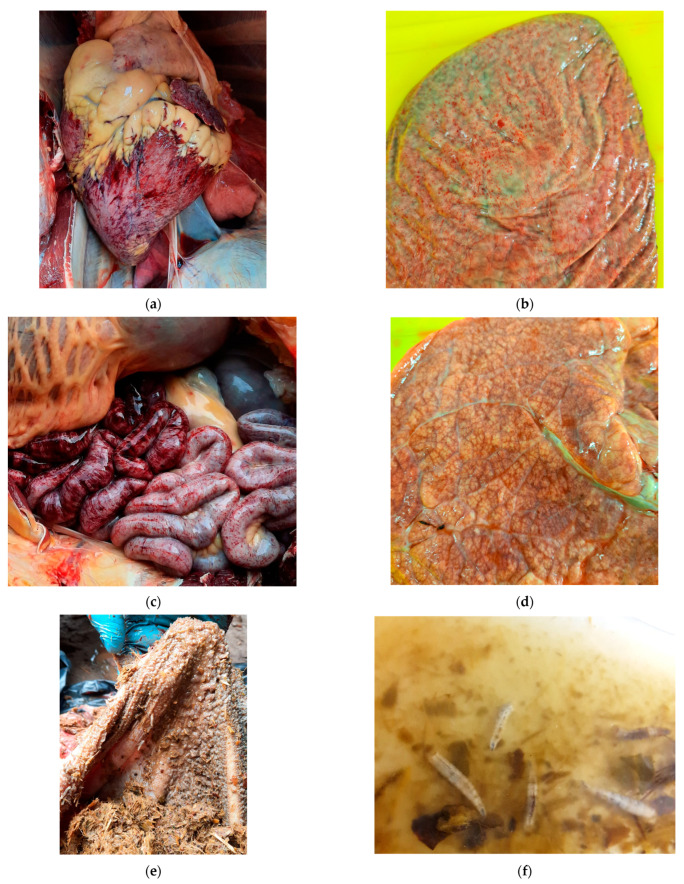
Necropsy findings in a 9-year-old European bison cow which died in an enclosure of Wolin National Park, Poland: (**a**) hemorrhages in the epicardium; (**b**) hemorrhages under the splenic capsule; (**c**) hemorrhagic enteritidis; (**d**) multifocal pulmonary emphysema; (**e**,**f**) the blowfly *Lucilia caesar*’s larvae in the rumen and jejunum contents.

**Figure 5:**
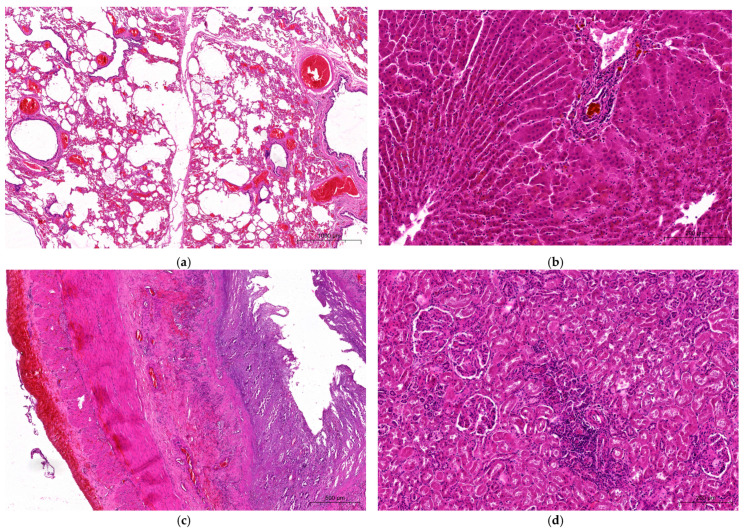
Microscopic examination of tissue sections from a 9-year-old European bison cow which died in an enclosure of Wolin National Park, Poland: (**a**) lung showing alveolar dilation and fragmentation of the alveolar walls adjacent to the areas of collapsed alveoli, with significant dilation and congestion in the pulmonary blood vessels. Hematoxylin and eosin (HE) staining, scale bar = 1000 µm; (**b**) liver showing the dissociation of hepatic cords with loss of hepatic cord architecture and the bile ducts expanded by variably sized accumulations of brown bile pigment. HE staining, scale bar = 200 µm; (**c**) small intestine showing extensive necrosis of the intestinal mucosa, with submucosal and subserosal hemorrhaging. HE staining, scale bar = 500 µm; (**d**) kidney showing mild lymphoplasmacytic interstitial nephritis. HE staining, scale bar = 200 µm.

External examination of the carcass revealed emaciation (carcass weight 300–320 kg) and generalized dehydration. No edema in the head, nor any of the changes in the nostrils, oral mucosa, limbs, hooves, or udders were observed, which are quite characteristic in BTV infections. However, at the necropsy there were detectable characteristic petechiae under the epicardium (Figure 4a) and on the splenic capsule (Figure 4b), severe hemorrhagic abomasitis and enteritis filled with bloody ingesta (Figure 4c), and multifocal pulmonary emphysema (Figure 4d), suggesting some hemorrhagic disease. Other observations included fly larvae present in the contents of the rumen and jejunum (Figure 4e), which were later genetically identified as maggots of the blowfly (*Lucilia caesar*, Linnaeus, 1758) based on the cytochrome oxidase subunit I gene [24]. Microscopic examination also revealed multifocal alveolar lung emphysema characterized by the presence of irregularly distended alveoli and large sac-like air spaces resulting from ruptures of their walls, with spike-like stumps protruding into their lumina. In the vicinity of the emphysema foci, there were areas of atelectasis with collapsed alveoli and severely engorged blood vessels (Figure 5a). There was moderate disruption of the hepatic cord architecture, with the dissociation of hepatocytes and disorganization of sinusoids mostly affecting the midzonal and periportal regions of the hepatic lobules. Occasionally, bile canaliculi and ducts contained luminal, globular, brown-to-bright-yellow material (bile) (Figure 5b). Despite marked autolysis of the intestinal mucosa, partial-to-full-thickness mucosal necrosis with hemorrhaging, fibrin, and eosinophilic cellular and karyorrhectic debris was observed throughout the entire length of the small intestine and colon. Occasionally, the remaining small intestinal crypts were ectatic, lined by attenuated epithelium, and contained sloughed epithelial cells admixed with moderate numbers of macrophages, lymphocytes, plasma cells, and a smaller number of intact and necrotic neutrophils. The inflammatory infiltrate composed mainly of lymphocytes and plasma cells extended through the muscularis mucosa into the submucosa. The submucosa and serosa were markedly expanded by abundant hemorrhaging (Figure 5c). In the kidneys, there were mild-to-moderate multifocal aggregates of lymphocytes and plasma cells adjacent to the renal tubules (Figure 5d).

#### 3.1.2. BTV Detection

Both the blood and spleen samples of the dead European bison cow tested positive for BTV in the pan-BTV real-time RT-PCR, with threshold cycle (C_t_) values of 23.8 (β-actin 21.1) and 24.2 (β-actin 20.3), respectively. Typing of the BTV with the commercial kit revealed the presence of BTV type 3 (C_t_ = 22.1 in blood samples and 23.5 in spleen homogenate; C_t_ for GAPDH = 22.8).

**Figure 6:**
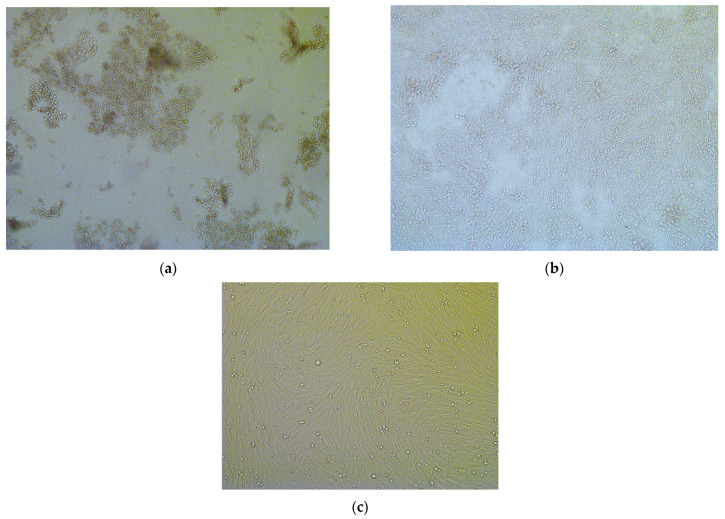
The cytopathic effects of BTV-3 isolated in BHK-21 cells from blood cells (**a**) and spleen (**b**) of a 9-year-old European bison cow which died in an enclosure of Wolin National Park, Poland, (passage 3) compared to non-infected cells (**c**).

Furthermore, BTV was also isolated in BHK-21 cell monolayers from the European bison cow’s blood cells and spleen homogenate. Three subsequent passages were performed. Some CPE was observed during the second and third passages (Figure 6a,b); however, the effect of the virus was much more pronounced in full-blood samples (Figure 6a). Bluetongue virus replication in BHK-21 cells was confirmed by the real-time RT-PCRs for blood cells (C_t_ = 29.3 and 13.4 during passages 2 and 3) and spleen (C_t_ = 36.3 and 27.6 during passages 2 and 3).

The involvement of other pathogens was excluded, as most test results were negative, except those for the test that revealed the presence of antibodies against SBV.

#### 3.1.3. *Culicoides* spp. Activity and BTV Detection

**Figure 7:**
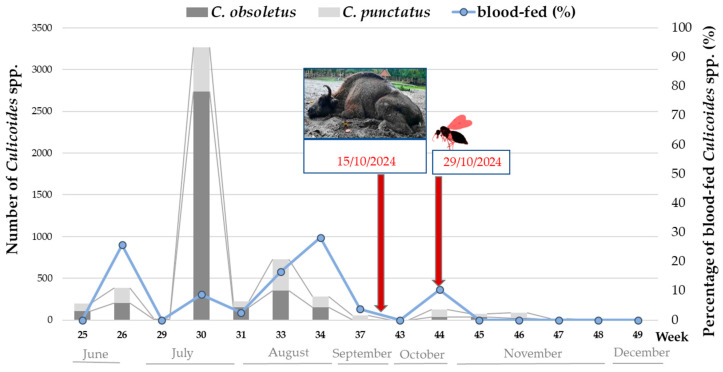
Timeline of *Culicoides* activity at Wolin National Park, Poland. The gray columns represent the abundance of the two most common midge species: *C. obsoletus* and *C. punctatus*. The blue dots connected with a line represent the percentage of blood-fed insects (secondary axis on the right), meaning the proportion of females that had a blood meal in their abdomen out of all the females caught at each time point. Red arrows indicate when BTV-3 was confirmed in a 9-year-old European bison cow and in a pool of blood-fed *Culicoides punctatus,* which were caught two weeks later.

A total of 5553 *Culicoides* individuals were caught during 15 night catches in the European bison enclosure at the WNP between June and December 2024. The most frequent were *C. obsoletus* (*n* = 3833) and *C. punctatus* (*n* = 1636). Other species included *C. achrayi* (*n* = 5), *C. circumscriptus* (*n* = 2), *C. fascipennis* (*n* = 3), *C. festivipennis* (*n* = 1), *C. furcillatus* (*n* = 1), *C. grisescens* (*n* = 2), *C. newsteadi* (*n* = 27), *C. pallidicornis* (*n* = 2) *C. pulicaris* (*n* = 39), and some unidentified species. Most of them were females of different gonotrophic forms (nulliparous, *n* = 2809; parous, *n* = 2021; blood-fed, *n* = 614; gravid, *n* = 85), with a small proportion of males (*n* = 33). The midges were most active in June and August (Figure 7); however, based on the presence of blood-fed females, the insects were actively feeding until the end of October. Thirty-three pools of blood-fed females were tested for BTV genetic material. Only one pool (3%) of 13 *C. punctatus* individuals, caught two weeks after the death of the European bison cow (Figure 7), was found to be BTV positive; the C_t_ in the PCR was 33.58. Typing of the BTV detected in *Culicoides* revealed the presence of BTV-3 (C_t_ = 32.8).

#### 3.1.4. BTV Strain Characterization

**Figure 8:**
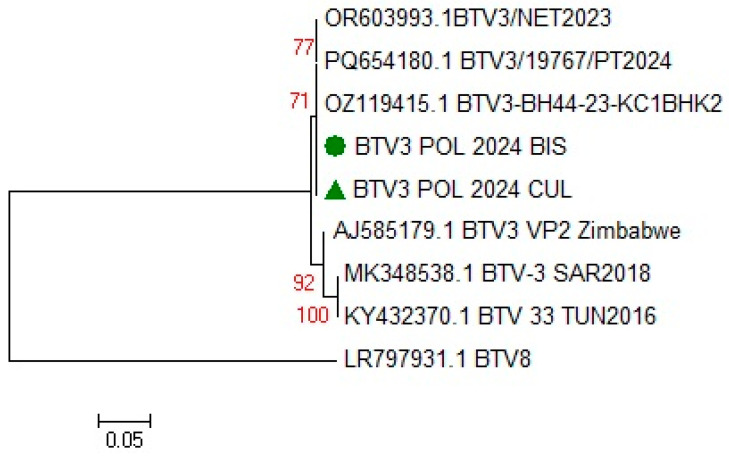
The phylogenetic relationships of the BTV-3 isolates detected in a 9-year-old European bison cow (●) who died in an enclosure at Wolin National Park, Poland, and a pool of *Culicoides* (▲) at the same park, with reference nucleotide sequences available in the GenBank database. Relationships are based on an 870 bp fragment of segment 2 of BTV-3. The phylogenetic tree was generated using the neighbor-joining method (Kimura2 parameter), as implemented in MEGA 5 software [24]. Bootstrap values (1000 replicates) over 70%, indicating significant support for the tree topology, are indicated next to the branches.

The phylogenetic analysis performed on an 870 bp fragment of segment 2 of the BTV-3 genome revealed a high nucleotide sequence similarity (99.7%) in the isolate from the European bison and the pool of *Culicoides* to BTV-3 strains detected in the Netherlands (the first BTV-3 isolate detected in Western Europe in 2023), Portugal (from a dog in September 2024) and Germany (GenBank accession no. OR603992, PQ654180, and OZ119415) (Figure 8). The nucleotide sequences of the Polish European bison and *Culicoides* BTV-3 were also 96.8% identical to the BTV-3 SAR2018 strain isolated from a sheep in Sardinia (Italy) in 2018 (GenBank accession no MK348538) and in a Barbarine ewe in Tunisia in November 2016 (GenBank accession no KY432370).

### 3.2. BTV Survey of European Bison in Poland

Antibodies to BTV were detected in 37 (23.1%) out of 160 European bison sampled in 2023 and 2024. Most seropositive animals were found in the northeastern free-ranging populations of the Białowieża and Knyszyńska Forests, and only a single reactor (a never-translocated 6-year-old healthy bull) was noted in the enclosure in Pszczyna in the south of the country (Table 1). The seroprevalence increased with age. Most seropositive animals were over 9 years of age. No BTV was detected in the blood of any of the seropositive European bison, except for the index case at the WNP described in Section 3.1.

## 4. Discussion

The appearance of BTV-3 infections in Poland is not surprising, as the disease had been reported in western Germany since the fall of 2023, but continuous monitoring there did not confirm cases of infection until the fall of 2024. This BTV-3 case, found as the first case in a European bison, was about 20 km away from the German outbreak registered in October 2024 [25], which implies that the virus most likely migrated with *Culicoides*. These midges are able to fly several kilometers over a few days by themselves, but a heavy wind can carry them hundreds of kilometers. The suspicion of BTV infection was reported to the Polish National Veterinary Research Institute, which has been involved in the monitoring of viral infections in the European bison population as part of a scientific collaboration with free-ranging population and captive herd managers since 2012 [19], while also being the National Reference Laboratory for BTV. Shortly thereafter, further outbreaks in domestic cattle (seven in 2024 on the western side of the country and three in 2025) were identified in the national monitoring program supervised by the General Veterinary Inspectorate (Figure S2). In early 2025, BTV unexpectedly appeared in the northeastern part of the country, which was likely related to the entry of BTV-3-infected animals. The virus is expected to spread further across the country in 2025. So far, however, in the majority of BT outbreaks in domestic ruminants, no clinical cases of BT have been identified. Despite the relatedness of European bison to domestic cattle, in which symptoms of BTV infection are rather less severe than in sheep, European bison appear to be more susceptible to infection with this virus. A relatively high mortality rate (30%) of BTV-8 infections was described in European bison in Germany in 2007 [19]. Clinical signs and mortality caused by BTV-3 have been observed in European bison in the Netherlands, Denmark, and Germany in the last two years [26].

The relatively severe course of BT in European bison is one concerning aspect of BTV-3’s circulation in Poland, and another is that, based on seroprevalence data, the arbovirus exposure rate in the species is higher than rates in other wild or farmed ruminants. This applies to *Culicoides*-borne BTV and SBV, as well as to tick-borne encephalitis virus (TBEV). Infections with BTV and SBV emerged in European bison in Poland simultaneously in 2012 [27]. While SBV has spread all over the country, BTV infections were limited almost exclusively to the northeastern populations [18]. The seroprevalence of SBV and BTV in European bison at the beginning of the epizootic reached 75 and 25%, respectively, and was significantly higher than in the cervids sampled at the same locations [27]. In this study, the presence of antibodies was also observed in the northeastern Białowieża and Knyszyńska Forest European bison populations in 2023–2024. Since no viremia has been detected in any of the seropositive bison, the serological evidence of infection is most probably associated with BTV-14, which was identified as circulating locally in cattle [28]. Its unexpected appearance in the east of the country was via transmission from over the eastern border [28,29,30]. Bluetongue virus serotype 8, which emerged in western Europe, has never reached Poland. The circulating BTV-14 was characterized by a very low pathogenicity, and the contamination of some illegally used vaccine was suspected as a source of infection, as the virus was probably attenuated and closely related to the reference BTV-14 strain [28,29]. The presence of antibodies in calves and European bison older than 9 years suggests either maternal immunity or the persistence of antibodies, as no virus was detected. Nevertheless, some limited circulation of BTV-14 in the European bison population could not be excluded, despite there being no evidence of it in the official monitoring of domestic ruminants.

The high tropism of arthropods to the species was also revealed in a TBEV serosurvey, where seroprevalence reached 63%. This high prevalence made the European bison a more sensitive indicator species than deer, which are considered sentinels of this zoonotic virus in the endemic foci [31]. Two factors should be considered relevant: firstly, due to their size and emission of large amounts of CO_2_ and other gases and odors, European bison are more attractive to insects; secondly, European bison naturally inhabit humid forests, which provide an excellent habitat for arthropod vectors to thrive in. With global warming, the insect activity season can be significantly extended, as observed in this study, where *Culicoides* spp. were feeding on hosts until late October and were active until November and perhaps even longer, given the effectiveness of the trap used. Previously, we observed that the number of *Culicoides* biting midges caught in the European bison reserve in the Białowieża Forest outnumbered those insects collected in the neighboring cattle farms several dozen times [18]. We have observed a change in tick activity and their feeding patterns on European bison skin from seasonal to year-round [32]. With prolonged arthropod activity, the risk of exposure to arthropod-borne pathogens also increases, because prevention of ectoparasite invasion is limited and highly inefficient in wild animals. Our study is the first to provide evidence of BTV-3 vector competence in one of the more abundant species of *Culicoides* in Europe, *C. punctatus*, the second most numerous species caught at the location of the BTV-3 outbreak in the European bison. In a recent study, Voight et al. [5] detected BTV-3 RNA in a pool of midges of undetermined parity status and mixed species, comprising *C. obsoletus*, *C. scoticus*, and *C. chiopterus*. However, since 1603 pools were tested in total, monitoring for the presence of the virus in the vector has a limited predictive value for BTV-3 circulation, as concluded by the researchers who made the detections. The effectiveness of such detection may increase if only parous and/or blood-fed midge pools are tested, as in our case. However, here again, BTV-3 could not be detected earlier, but only after a case occurred in the European bison. Nevertheless, the virus was observed to circulate even two weeks later, which suggests further transmission that will be verified by continuation of entomological monitoring in 2025. Parous *C. punctatus* have been demonstrated to contain BTV-1 RNA in Italy: in Sardinia in 2013 [33] and in Abruzzo and Apulia in 2014 [34]. The species was reported to possibly be involved in BTV transmission in Turkey in 2007 [35] and in a previous BTV-8 epidemic in Germany [36]. In the latter study, extant *Culicoides* activity was also observed in the winter of 2007/2008, which may explain the possibility of BTV overwintering in the insect vector.

To conclude, it is difficult to predict for the time being the impact of BTV-3 on the protection of this iconic species, one of the major symbols of rewilding initiatives and biodiversity conservation in Europe. It is important that we begin to look at climate risks comprehensively, taking into account the health of the environment as part of the well-being of the ecosystem and the humans living in it, which together constitute OneHealth.

## Figures and Tables

**Table 1:** Descriptive statistics of European bison exposure to BTV based on serology results for the presence of specific antibodies followed by RT-PCR for detectable RNAemia of seropositive animals.

Variable	BTV Seropositive		Pan-BTV RT-PCR	
	*n*/*N* ^1^	% (CI ^2^)	*n*/*N* ^1^	Strain
**Origin (total population size ^3^)**				
A—Wolin National Park (9)	1 ^4^/3	33.3 (0.8–90.6)	1 ^4^/1	BTV-3
B—Zachodniopomorskie herds (349)	0/4	0 (0–60.2)	0/0	
I—Bieszczady (750)	0/10	0 (0–30.8)	0/0	
C—Poznań Zoo (9)	0/1	0 (0–97.5)	0/0	
D—Gołuchów (7)	0/2	0 (0–84.1)	0/0	
E—Pszczyna (53)	1/51	2 (0.05–10.5)	0/1	
F—Niepołomice (16)	0/1	0 (0–97.5)	0/0	
G—Gdańsk Zoo (8)	0/2	0 (0–84.1)	0/0	
H—Bałtów (9)	0/4	0 (0–60.2)	0/0	
J—Borecka Forest (127)	0/3	0 (0–70.8)	0/0	
K—Augustowska Forest (23)	0/1	0 (0–97.5)	0/0	
L—Knyszyńska Forest (298)	3/16	18.8 (4.0–45.6)	0/3	
M—Kopna Góra	0/1	0 (0–97.5)	0/0	
N—Białowieża Forest (829)	33/62	53.2 (40.1–66.0)	0/33	
**Population type**				
Free-ranging	35/82	42.7 (31.8–54.1)		
captive	3/79	3.8 (0.7–10.7)		
**Age group**				
≤1 year old	2/26	7.7 (1.0–25.1)	0/1	
2–3 years old	1/38	2.6 (0.056–13.8)	0/1	
≥4 years old	34/97	35.0 (25.9–45.8)	1 ^4^/34	BTV-3
**Gender**				
Female	22/72	30.6 (20.5–43.0)	1 ^4^/22	BTV-3
Male	15/89	16.8 (9.7–26.3)	0/15	
**Health status**				
Immobilized (healthy)	7/84	8.3 (3.4–16.4)	0/7	
Eliminated	17/40	42.5 (27.0–59.1)	0/17	
Fallen	11/26	42.3 (23.4–63.1)	1 ^4^/11	BTV-3
Dead in traffic accident	2/7	28.6 (3.7–71.0)	0/2	
Missing data	1/4	25.0 (0.6–80.6)	0/1	

^1^ number of seropositive European bison/all tested bison (missing data were excluded); ^2^ binomial exact 95% or one-sided 97.5% confidence interval; ^3^ according to the most recent data from 2022 [15]; ^4^ this is the BTV-3 case described in this case report. Significant differences are marked in bold (*p* < 0.05).

## Data Availability

Data are available from Magdalena Larska (National Veterinary Research Institute, Puławy, Poland) upon request.

## Supplementary Materials

The following supporting information can be downloaded at: https://www.mdpi.com/article/10.3390/pathogens14040377/s1, Figure S1: Numbers by country and distribution of European bison (*Bison bonasus*) populations worldwide registered in the European Bison Pedigree Book 2023 [15]. Figure S2: The distribution of BTV-3 cases in cattle in Poland recorded until 1 March 2025, by the Central Veterinary Office (the map is available at: https://bip.wetgiw.gov.pl/bt/mapa/, accessed on 27 February 2025).
