# Supplementary material for: First Detection of Bluetongue Virus Type 3 in Poland in 2024—A Case Study in European Bison (Bison bonasus)
Source: Pathogens. 2025 Apr 12;14(4):377. doi: 10.3390/pathogens14040377 (PMC12030162; doi:10.3390/pathogens14040377)
Supplement: Supplementary file 1 [file pathogens-14-00377-s001.zip › pathogens-3540620-supplementary.pdf]

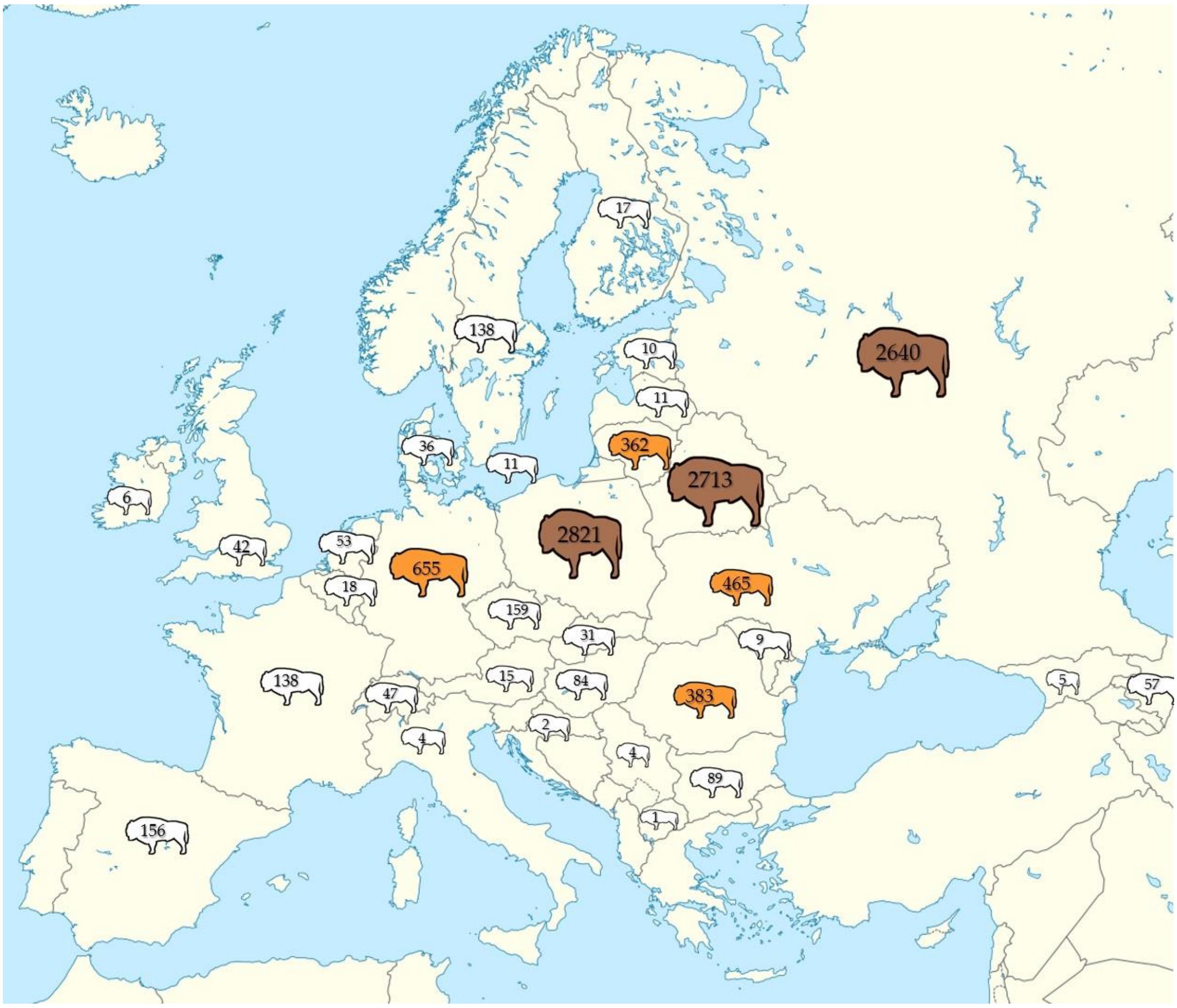

Figure S1. Numbers by country and distribution of European bison (*Bison bonasus*) populations in Europe registered by the European bison Pedigree Book 2023 [15]. Additionally, six European bison are reared in two locations in Indonesia. The size of the is intended to reflect approximately the size of the population. In addition, the largest populations are shown in brown and slightly smaller populations in orange.

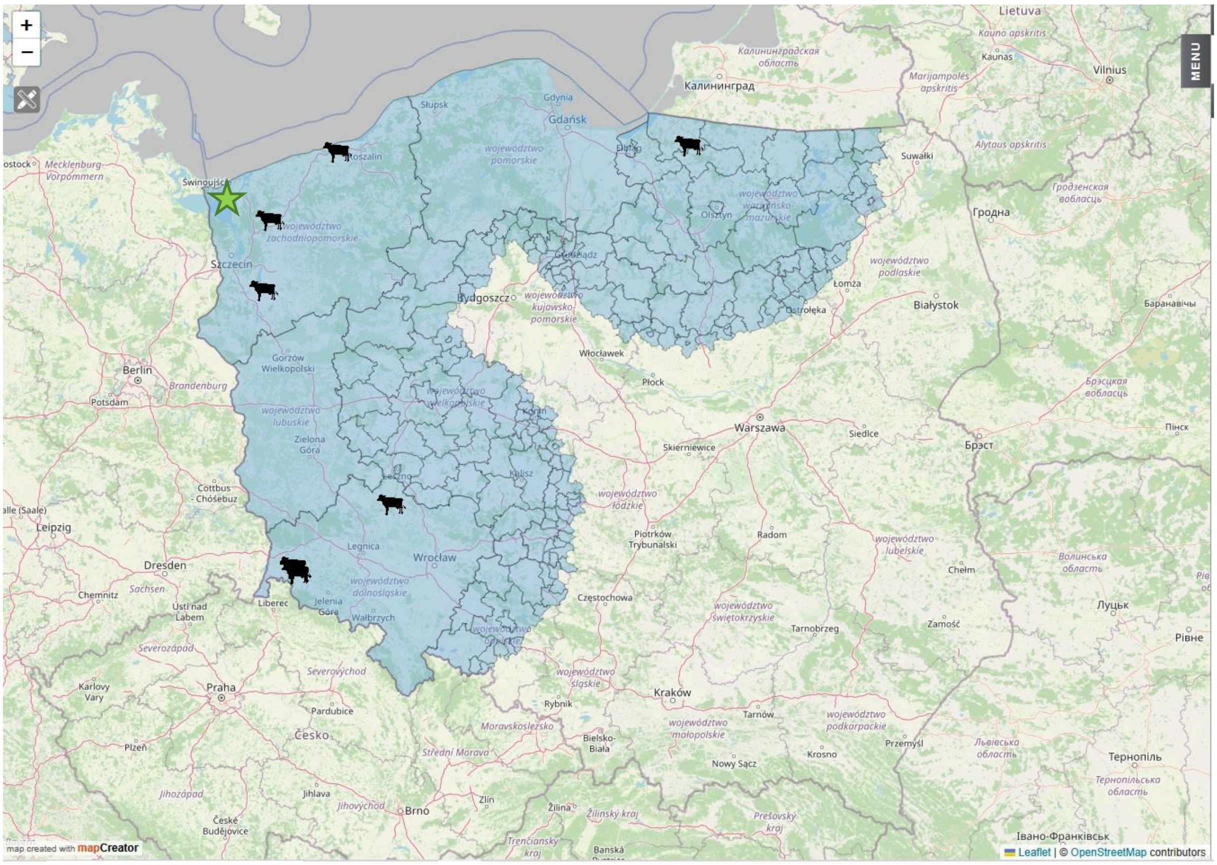

Figure S2 The distribution of BTV-3 cases in cattle in Poland recorded until March 25, 2025 by the Central Veterinary Office at the map available at: https://bip.wetgiw.gov.pl/bt/mapa/. The black and red cow figures represent the locations of the outbreaks in 2024 and 2025, respectively, while the case of the infected European bison is marked with a green asterisk. The blue areas indicate the restriction zones.
